# The Relation Between Discipline Identity and Academic Achievement Within a Marketized Higher Education Context: A Serial Mediation Model of Approaches to Learning and Course Complaints

**DOI:** 10.3389/fpsyg.2022.749436

**Published:** 2022-09-27

**Authors:** Louise Taylor Bunce, Melanie Bennett, Siân E. Jones

**Affiliations:** ^1^Psychology Department, University of Winchester, Winchester, United Kingdom; ^2^Department of Psychology, Goldsmiths College, University of London, London, United Kingdom; ^3^Division of Psychology, Sociology and Education, Queen Margaret University, Edinburgh, United Kingdom

**Keywords:** approaches to learning, academic achievement, discipline identification, complaining, social identity

## Abstract

Social-psychological dimensions of learning are under-researched, but they affect student achievement. Within a marketized higher education context in England, United Kingdom, this study examined whether the relation between students’ social identities as members of their discipline and academic achievement could be further understood by considering the mediating roles of approaches to learning and frequency of making course complaints. Undergraduates (*N* = 679) completed a questionnaire to assess these constructs. As expected, approaches to learning and course complaining both acted as serial mediators of the link between discipline identification and academic achievement: stronger discipline identification was related to more deep approaches to learning, less complaining, and higher achievement, whereas weaker discipline identification was related to more surface approaches to learning, more complaining, and lower achievement. The findings suggest that addressing these social-psychological aspects of learning could improve students’ academic achievement.

## Introduction

Understanding the factors that influence students’ academic success remains of critical significance for educators. It is now accepted that a full understanding of the processes that lead to successful learning requires consideration of not only individual psychological factors, but also of social-psychological dimensions as well (Platow et al., 2017). Although learning is a highly contextual process that typically occurs within a social context, this aspect is often overlooked (Platow et al., 2017). In the current study, we considered academic achievement within the context of the marketization of higher education and an increasing complaints culture (Mitchell, 2019) in England, United Kingdom. We adopted a social identity approach, testing a serial mediation model to examine the impact of three social-psychological variables on academic achievement: students’ social identification with other students in their discipline or “discipline identity,” approaches to learning, and frequency of making course complaints. Specifically, the model assessed the possible combined impacts of approaches to learning and complaining frequency on the positive relation between discipline identity and academic achievement. These findings will have important implications for demonstrating the relevance of social-psychological variables on academic achievement and for understanding how to improve academic achievement in a marketized higher education context.

### Marketization of Higher Education in England

Approximately 20 years ago, the British government transferred responsibility for the cost of higher education tuition in England away from the taxpayer and onto individual students (Dearing, 1997). This move was in line with the neoliberalist agenda, which views higher education not as a societal good but as an individual private one. This process defines students as “consumers” and has transformed universities into service providers that focus on marketing, recruitment, and “customer” satisfaction metrics (Williams, 2013). While the majority of students seems to have resisted this new identity (Taylor Bunce et al., 2022), marketization has, nonetheless, had a negative impact on learning and teaching. For example, it has placed pressure on staff to attain student satisfaction (potentially at the expense of learning), encouraged students to pursue their consumer rights (Senior et al., 2017; Jabbar et al., 2018; Wong and Chiu, 2019; King and Bunce, 2020), and increased student complaints (Mitchell, 2019). In addition, it has been associated with a decrease in academic achievement among students who adopt a stronger consumer identity (Bunce et al., 2017).

### Discipline Identity in Higher Education

The creation of a student as consumer identity and its subsequent impact on learning and teaching seems to be in conflict with the more traditional identity of a student as a member of a community of scholars in their discipline. A study by Bliuc et al. (2011a,b) was among the first to examine potential relations between students’ social identification with other students in their discipline or “discipline identity” and its impact on students’ learning approaches and achievement. They adopted a social identity approach based on social identity theory (Tajfel and Turner, 1979), which proposes that when people identify strongly with a particular group, and their membership is salient, individuals are highly likely to behave in line with the norms of the group (Ellemers et al., 2002). Norms comprise attitudes and behaviors that motivate individuals to support the interests of the group (Haslam, 2017). Bliuc et al. thus examined whether students’ social identification with other students in their discipline—discipline identification—is a social-psychological variable that affects academic achievement. Their hypothesis was that a strong discipline identity would positively affect academic achievement because it would support effective attitudes toward studying and study behaviors. In their research set in a non-fee-paying context in Romania, they examined this hypothesis by considering the extent to which psychology students adopted deep or surface approaches to learning. These approaches to learning have been well established in the literature following their introduction by Marton and Säljö (1976) and development by Entwistle and Ramsden (1983) and Biggs (1987). Deep approaches to learning involve enjoying searching for meaning and engaging with ideas with the intention of understanding, whereas surface approaches involve superficial engagement with a focus on simply reproducing knowledge to avoid failure. Although the relation between learning approach and academic achievement is influenced by multiple individual and external factors (Eley, 1992; Karagiannopoulou et al., 2020; Mørk et al., 2020; Tuononen et al., 2020), there is a reliable and robust association between deep approaches and higher academic achievement (Richardson et al., 2012). There is also evidence of an association between surface approaches and lower academic achievement (e.g., Trigwell and Prosser, 1991; Richardson, 1994; Diseth and Martinsen, 2003; Amirali et al., 2004).

As hypothesized by Bliuc et al. (2011a), students’ approaches to learning were predicted by the strength of their discipline identity: students with a strong discipline identity were more likely to have goals and beliefs about learning that were consistent with a desire to understand and construct meaning, that is, to adopt a deep approach to learning. In contrast, students with a weaker discipline identity were more likely to adopt a surface approach to learning. Subsequently, Bliuc et al. found that a deep approach was associated with higher academic achievement, whereas a surface approach was associated with lower achievement.

### Approaches to Learning and Complaining in a Marketized Higher Education Context

There is emerging evidence suggesting that students’ approaches to learning are also negatively affected by the marketization of higher education, with its emphasis on extrinsic motivation for attending, such as obtaining well-paid employment (Naidoo and Williams, 2015). For example, Bunce and Bennett (2021) recently showed that approaches to learning mediate the negative relation between a consumer identity and academic achievement. Specifically, they found that students who identified more strongly as consumers had lower achievement because they were less likely to adopt deep approaches to learning and more likely to adopt surface approaches. Tomlinson (2017) found evidence of some students adopting instrumentalist approaches to learning, perceiving lectures as a passive form of entertainment, and evaluating their education in terms of value for money as opposed to other types of educational value (see also King and Bunce, 2020).

Requests for students to evaluate their higher education experience within a marketized context have become ubiquitous in order to improve service and satisfaction levels (Hammonds et al., 2017). It is perhaps not surprising, therefore, that there has been a steady rise in student complaints in England, particularly about value for money and “service issues,” such as perceived poor teaching quality and perceived difficult content (Mitchell, 2019). This could be because some students may feel entitled to receive a good degree in return for their tuition fees as opposed to earning a degree through intellectual effort (Finney and Finney, 2010; Tomlinson, 2017). This may lead to dissatisfaction and complaints about learning and teaching based on unrealistic expectations about the nature of learning in higher education. In turn, this could lead to students seeking explanations for content perceived as difficult based on external reasons, such as failure of teaching staff or poor services, as opposed to internal ones, such as lack of effort (Newman and Jahdi, 2009). Complaining, therefore, seems to situate students’ negative experiences with the service provider, or institution, rather than themselves.

The propensity to complain following unsatisfactory experiences is not an individual phenomenon, but one that is heavily dependent on identification with the relevant social group (Watkins and Liu, 1996). Because social groups prescribe behavior through group norms, if a group norm is one of dissatisfaction, then complaining will be supported by this norm. In contrast, when the group norm is one of satisfaction, a complainant may be labeled as a “whiner” and risk negative social consequences such as marginalization or exclusion from the group (Kowalski, 1996). To maintain valued group membership, potential complainers thus need to behave in line with group norms and be sensitive to their level of complaining.

The current study specifically examined the extent to which students complain when they are routinely asked to evaluate their courses (course complaining). Even though this is done anonymously and may not necessarily involve conforming to group norms, this everyday level of complaining is more likely to pertain to group norms than the relatively more rare and serious complaints that students can make through formal procedures. Given that students’ group membership and awareness of group norms shape their behavior, it follows that students’ complaining behavior will be influenced by the strength of their discipline identity and its associations with approaches to learning. Complaining will contradict group norms relating to deep learning, including embracing difficulty as an intellectual challenge rather than perceiving it as a barrier to progression. We argue here that a strong discipline identity, and its relation to deep approaches to learning, will work together to improve satisfaction with learning and thereby reduce complaining, which will ultimately be associated with higher achievement. In contrast, a weak discipline identity, and its relation to surface approach to learning, will work together to minimize satisfaction with learning and thereby increase complaining, which will ultimately be associated with lower achievement.

### Current Study

The current study sought to understand how academic achievement is affected by social-psychological variables relevant in a marketized higher education context: discipline identification, approaches to learning, and frequency of course complaining. Specifically, we tested two causal mediation models. The first model examined the hypothesis that stronger discipline identification is related to more deep approach to learning, that deep approach to learning is related to lower frequency of course complaining, and together, these variables predict higher academic achievement. The second model examined the hypothesis that weaker discipline identification is related to more surface approach to learning, that surface approach to learning is related to higher frequency of course complaining, and together, these variables predict lower academic achievement.

We controlled for demographic factors including age (mature versus other), gender (female versus other), and ethnicity (Black, Asian, or minority ethnic versus other), as well as grade goal (first class versus other), which have been associated with academic achievement in a marketized context (Richardson et al., 2012; Bunce et al., 2017).

## Materials and Methods

### Participants

Data were provided by 679 undergraduates (97% were full-time) at higher education institutions in England, United Kingdom, who were liable for the full cost of their tuition (up to £9,250 for home students). The average age was 21.6 years (*SD* = 5.56 years), and there were 409 women (60%), 266 men (39%), and four students who preferred not to answer (1%). Most students (578, 85%) described themselves as White, 40 (6%) described themselves as Black, 34 (5%) as Asian, and 27 (4%) as mixed ethnic background. A first class degree result was the grade goal for 292 (43%) of students. Students were from 99 different disciplines at 81 higher education institutions, of which 16% were research-intensive institutions as opposed to teaching-focused ones.

### Measures

In an online questionnaire, students first provided demographic information, including age, gender, and ethnicity. They then stated the classification of degree that they hoped to attain (grade goal) and reported the grade (expressed as a percentage) that they had received for their most recent assessment (academic achievement). The frequency with which students complained about their course (course complaining) was assessed by asking them to what extent they agreed with the item “I regularly complain when asked to give feedback on my course,” on a 5-point scale from strongly disagree (1) to strongly agree (5).

To measure discipline identification, we followed Bliuc et al. (2011a) and adapted the four item scale used by Doosje et al. (1995). The items were as: “I feel strong ties with other students who are studying my subject,” “I am pleased to be a student in my field of study,” “I identify with other students in my field of study,” and “I see myself as a student in my field of study.” Students rated their level of agreement with each item on a 5-point scale from strongly disagree (1) to strongly agree (5). Cronbach’s alpha indicated an acceptable reliability score of 0.76 for these items.

Finally, deep and surface approaches to learning were assessed using the Revised Study Process Questionnaire (Biggs et al., 2001). Students rated their level of agreement with 20 items on a 5-point scale (1 = strongly disagree and 5 = strongly agree), e.g., “I find that at times studying gives me a feeling of deep personal satisfaction” (deep approach) and “My aim is to pass the course while doing as little work as possible” (surface approach). Cronbach’s alpha indicated acceptable reliability scores of 0.73 for deep approach to learning items and 0.71 for surface approach to learning items.

### Procedure

Participants were recruited through on-campus advertising at the authors’ institutions as well as *via* posts on social media targeted at student groups. The study was described as assessing students’ attitudes toward their university education. It was part of a larger study, with aspects of the data previously published in Bunce and Bennett (2021). If participants gave consent by ticking a box at the start of the online questionnaire, they worked through the questionnaire as described above, with each set of questions presented on a new page. The questionnaire took approximately 10 min to complete. Ethical approval was obtained from the first author’s institution before data collection began.

## Results

### Data Screening

Prior to analysis, the data were checked for outliers, patterns in missing values, and violations of assumptions for parametric data. Univariate outliers were removed in the appropriate analyses to ensure that they did not disproportionately influence the results.

Descriptive statistics and bivariate correlations among the key dependent variables are given in Table 1. Initially, four variables were added as covariates because they had significant effects on either the mediators or the outcome variable: age, gender (female versus other), ethnicity (white versus other), and grade goal (first class versus other). However, running the models without these covariates showed no meaningful differences in the findings on any path direction. Thus, for simplicity, models without the covariates are reported.

**Table 1:** Correlations, means, and standard deviations (SD) for key variables.

Mean (SD)	1	2	3	4	5
	67.38 (10.34)	2.62 (0.95)	3.84 (0.77)	3.64 (0.65)	2.38 (0.76)
Academic Achievement	–				
Course Complaining	−0.126^***^	-			
Discipline Identification	0.090^*^	−0.137^***^	–		
Deep Approach	0.214^***^	−0.162^***^	0.321^***^	–	
Surface Approach	−0.140^***^	0.150^***^	−0.182^***^	−0.381^***^	**-**

* *p* < 0.05; ^**^p < 0.01;

*** *p* < 0.001.

### Serial Mediation

PROCESS Model 6 was employed to test the hypotheses that Deep Approach to Learning (Hypothesis 1) or Surface Approach to Learning (Hypothesis 2) and Course Complaining would mediate in series the link between Discipline Identification and Academic Achievement. Deep or Surface Approach to Learning and Course Complaining were included as serial mediators of the path from Discipline Identification (the independent variable) to Academic Achievement (the outcome variable). The aim of the subsequent reported analysis was to examine the hypothesized impact of discipline identification on academic achievement and to explore whether deep (1) or surface (2) approach to learning and course complaining mediated in series the relation between these two variables.

#### Deep Approach to Learning

**Figure 1:**
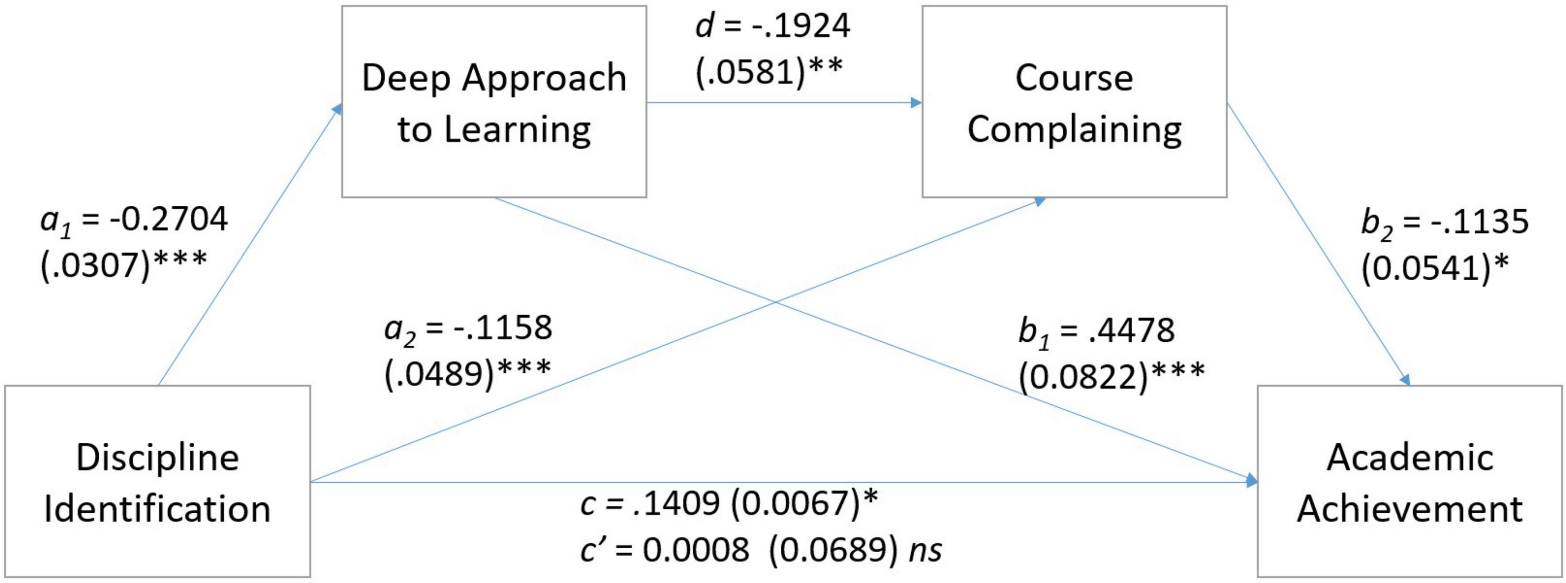
Serial mediation of deep approach to learning and course complaining on the relation between discipline identification and academic achievement (standard errors are shown in parentheses). ^*^p < 0.05, ^**^p < 0.01, and ^***^p < 0.001.

Analysis by PROCESS Model 6 (Hayes, 2018) using 5,000 bootstrap samples tested whether Deep Approach to Learning and Course Complaining would provide indirect paths between Discipline Identification and Academic Achievement (see Figure 1). Although there was no direct effect of Discipline Identification on Academic Achievement, there were three significant indirect paths between Discipline Identification and Academic Achievement. These were (a) *via* both Deep Approach to Learning and Course Complaining, *B* = 0.0059, *SE* = 0.0036, LLCI = 0.0004, ULCI = 0.0140, (b) *via* Deep Approach to Learning alone, *B* = 0.1211, *SE* = 0.02470255, LLCI = 0.0737, ULCI = 0.1738, and (c) *via* Course Complaining alone, *B* = 0.0131, *SE* = 0.0088, LLCI = 0.0001, ULCI = 0.0338. In other words, course complaining and deep approach to learning each independently mediated the link between discipline identification and academic achievement. As well as this, when placed in series, there was a significant path from discipline identification to deep approach to learning to course complaining to higher academic achievement. Through this path, deep approach to learning were negatively associated with course complaining, meaning that more deep approaches were associated with less course complaining. It is also worth highlighting the new finding of a direct negative relation between course complaining and academic achievement whereby less course complaining was related to higher achievement.

Two significant contrast effects were revealed, showing that the path *via* both Deep Approach to Learning and Course Complaining was significantly stronger than the path *via* Deep Approach to Learning alone, *B* = 0.115, *SE* = 0.0256, LLCI = 0.067, ULCI = 1,681. Additionally, the path *via* Deep Approach to Learning alone was significantly stronger than the path *via* Course Complaining alone, *B* = 0.1080, *SE* = 0.0277, LLCI = 0.0549, ULCI = 0.1651.

#### Surface Approach to Learning

**Figure 2:**
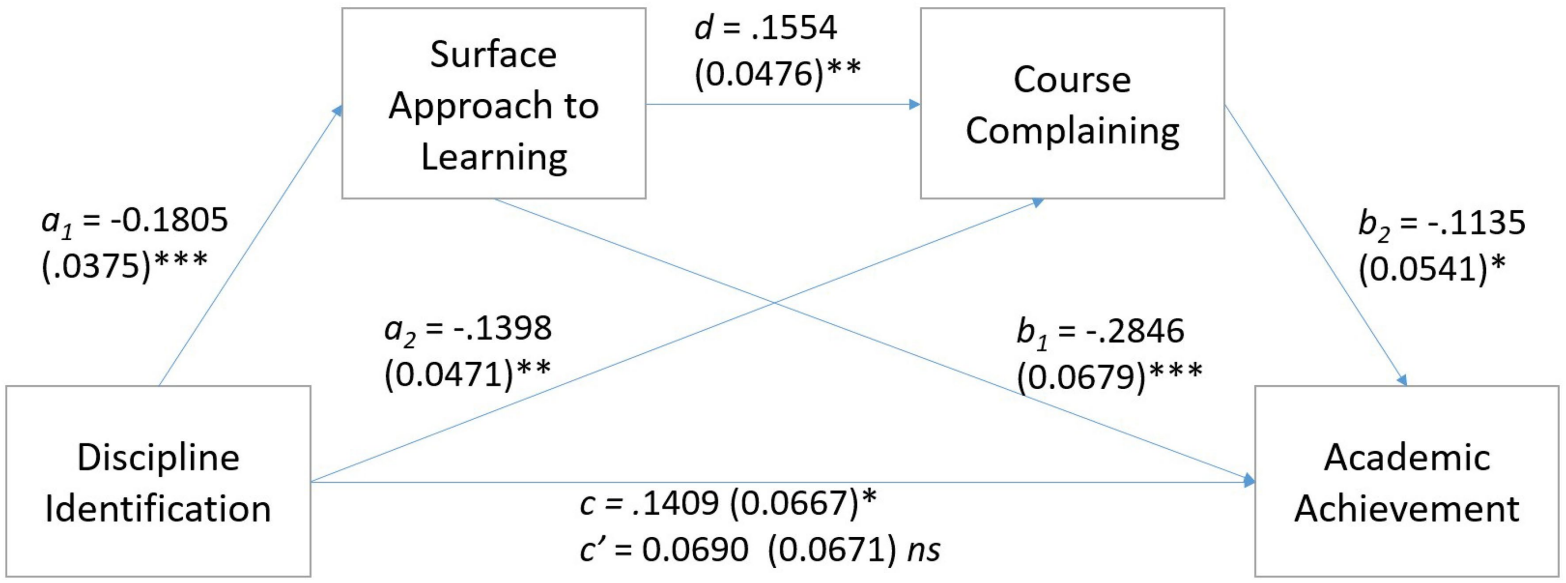
Serial mediation of surface approach to learning and course complaining on the relation between discipline identification and academic achievement (standard errors are shown in parentheses). ^*^p < 0.05, ^**^p < 0.01, and ^***^p < 0.001.

Analysis by PROCESS Model 6 (Hayes, 2018) using 5,000 bootstrap samples tested whether Surface Approach to Learning and Course Complaining would provide indirect paths between Discipline Identification and Academic Achievement (see Figure 2). Although there was no direct effect of Discipline Identification on Academic Achievement, there were three significant indirect paths between these two variables. These were (a) *via* both Surface Approach to Learning and Course Complaining, *B* = 0.034, *SE* = 0.0021, LLCI = 0.0002, ULCI = 0.0086, (b) *via* Surface Approach to Learning alone, *B* = 0.0514, *SE* = 0.0178, LLCI = 0.0216, ULCI = 0.0904, and (c) *via* Course Complaining alone, *B* = 0.0171, *SE* = 0.0101, LLCI = 0.0007, ULCI = 0.0403. In other words, course complaining and surface approach to learning each independently mediated the link between discipline identification and academic achievement. When placed in series, there was also a significant path from discipline identification to surface approach to learning to course complaining to academic achievement. Through this path, surface approach to learning was positively associated with course complaining whereby more surface approach was associated with more complaining.

There was one significant contrast effect. The path *via* both Surface Approach to Learning and Course Complaining was significantly stronger than the path *via* Surface Approach to Learning alone, *B* = 0.0479, *SE* = 0.0176, LLCI = 0.0187, ULCI = 0.0861.

## Discussion

By exploring how students’ social identification with their discipline, approaches to learning, and course complaining are related to academic achievement, this study extended an emerging body of research showing the importance of social-psychological factors for learning. More specifically, this research was set in a marketized higher education context to explore a serial mediation model testing whether the positive relation between discipline identification and academic achievement could be further understood in relation to students’ approaches to learning and frequency of course complaining. We tested the hypotheses that stronger discipline identification would support more deep approaches to learning, less complaining, and higher achievement, whereas weaker discipline identification would support more surface approaches to learning, more complaining, and lower achievement. These hypotheses were supported; thus, we replicated and extended previous research showing that there are relevant and important associations between social-psychological factors and academic achievement.

### Course Complaining

Finding evidence of a direct negative relation between course complaining and academic achievement is novel and highly relevant within a marketized higher education context, which has seen a rise in the level of student complaints (Newman and Jahdi, 2009; Mitchell, 2019). It builds on prior work by Bunce et al. (2017) on the negative impact of a consumer identity on achievement and suggests that complaining may be an additional relevant aspect of a consumer identity that has implications for student outcomes.

As predicted, course complaining was affected by the social-psychological variable of discipline identification and its associations with approaches to learning. We found that students with a strong discipline identity were less likely to complain, which may be for two reasons. First, they may be more likely to find learning intrinsically satisfying (in line with deep approaches) and have less cause to complain, such as over perceived difficult content. Alternatively, if they are dissatisfied, they may be less likely to complain because complaining risks marginalization or social exclusion if it is not in line with relevant group norms for behavior (Kowalski, 1996), in this case norms that support deep rather than surfaces approaches to learning. Furthermore, course complaining provided significant indirect paths between discipline identification and academic achievement, both alone and combined with approaches to learning. Again, this further demonstrates the relevance of social-psychological variables on behaviors that affect students’ academic achievement. These findings emphasize the importance of understanding students’ perceptions of group norms relating to expressions of satisfaction/dissatisfaction (Hogg and Reid, 2006), particularly in a marketized higher education context (Bunce, 2019; Lygo-Baker et al., 2019).

### Approaches to Learning

Finding that discipline identification was both positively related to deep approach to learning and academic achievement, and negatively related to surface approach and academic achievement, replicates a number of studies (e.g., Bliuc et al., 2011a,b; Smyth et al., 2015). Our findings add weight to the argument that the way students approach learning can be predicted by variables that reflect a student’s sense of belonging with other students in their discipline. The indirect effect of discipline identification on academic achievement *via* deep approach demonstrates that a course-specific learning context that fosters a strong discipline identity contributes to improved academic achievement.

According to our measure of deep approach to learning, students with a stronger discipline identity were more likely to study with the intention of developing an understanding of the material and constructing meaning, compared to students who have a weaker discipline identity. This is consistent with social identity research on the predictive value of strong social identities in relation to behaviors that are highly relevant to that specific social identity (e.g., Haslam et al., 2001; Cameron, 2004; Bliuc et al., 2007). Similarly, the link between deep approach to learning and academic achievement emphasizes the importance of improving learning outcomes by teaching in ways that foster deep approaches to learning. Again, as found by Bliuc et al. (2011a), discipline identification was not directly related to academic achievement, indicating the importance for future research to test alternative mediators and moderators to this relation. For example, some research suggests that there may discipline differences in students’ approaches to learning (Nelson Laird and Garver, 2010).

### Limitations and Future Research

This study has limitations that need to be addressed in future research. First, the sample is likely to represent students who were more engaged with their education, given their willingness to complete the questionnaire voluntarily. In addition, the sample diversity was narrow, with an underrepresentation of male students and students from ethnic minority groups. Complaining frequency was measured in relation to the less serious everyday level of complaints about the students’ own courses, but it would be important to assess different types of complaining behavior that vary in the extent to which they are done publicly or privately. For example, complaining to a fellow student representative is more public and potentially more dependent on group norms than complaining through an anonymous questionnaire. It would also be important for future work to consider further the nature of complaints made by students, such as those relating to differences in complaints regarding educational processes (e.g., organization or time taken to receive feedback on assignments) and educational content (e.g., perceived difficult content or challenging assignment briefs). It could be that students with a stronger discipline identity complain less about content than students with a weaker discipline identity, but discipline identification may not be related to complaints about educational processes. This could be examined in future work to explore further the relations between discipline identification and complaining behavior.

The cross-sectional nature of the research is also limiting in that the constructs tested (i.e., discipline identification, course complaining, and approaches to learning) are, in themselves, situationally dependent variables that are likely to change over time and in response to different course demands. Relations may also be reciprocal, with learning behaviors influencing discipline identification and discipline identification influencing learning behaviors, as found by Platow et al., (2013). Smyth et al. (2015) also suggested that academic achievement may impact discipline identity whereby performing well on a course reinforces social identification with the discipline.

Possible ways of strengthening students’ discipline identity represent another relevant direction to pursue in further research aimed at enhancing the quality of students’ engagement with their learning. Finding that social identity processes are implicated in academic achievement lends theoretical support to student activities (such as team-building days and discipline-specific student societies) that aim to foster group cohesion and instill group norms (e.g., Jetten et al., 1997). Our research suggests that these aspects of a course might drive the group processes that enhance student learning and should be integral to course design. Determining the types of activities that consolidate a strong discipline identity, and their resultant effects on academic achievement are central avenues for future research.

## Conclusion

While research has shown that discipline identification provides a meaningful way to understand approaches to learning and academic achievement, the current study revealed additional relevant relations with student complaining within a marketized higher education context. Our findings demonstrate that complaining affects students’ academic achievement because it is affected by social identity processes: students with a strong discipline identity were less likely to complain because they adopted more deep approaches to learning whereby learning is approached as an enjoyable and inherently satisfying process. In contrast, students with a weak discipline identity were more likely to complain because they adopted more surface approaches to learning whereby learning is approached superficially and as a means to an end. At a more applied level, our findings emphasize that student complaining is not merely an individual phenomenon, but one dependent on social-psychological factors. Educators should seek to enhance a sense of discipline identification among students, with its associated experience of learning being intrinsically satisfying, because this is ultimately associated with less complaining and improved academic achievement.

## Data Availability Statement

The datasets presented in this article are not readily available because ethical approval was not obtained from participants to share the data. Questions regarding the datasets should be directed to the first author.

## Ethics Statement

The studies involving human participants were reviewed and approved by the University of Winchester, Department of Psychology Ethics Committee. The patients/participants provided their written informed consent to participate in this study.

## Author Contributions

SJ helped with analysis and writing. MB helped with design, recruitment, and analysis. All authors contributed to the article and approved the submitted version.

## Conflict of Interest

The authors declare that the research was conducted in the absence of any commercial or financial relationships that could be construed as a potential conflict of interest.

## Publisher’s Note

All claims expressed in this article are solely those of the authors and do not necessarily represent those of their affiliated organizations, or those of the publisher, the editors and the reviewers. Any product that may be evaluated in this article, or claim that may be made by its manufacturer, is not guaranteed or endorsed by the publisher.
